# Characterization and Comparative Analysis of Complete Chloroplast Genomes of Four *Bromus* (Poaceae, Bromeae) Species

**DOI:** 10.3390/genes15060815

**Published:** 2024-06-20

**Authors:** Shichao Li, Chunyu Tian, Haihong Hu, Yanting Yang, Huiling Ma, Qian Liu, Lemeng Liu, Zhiyong Li, Zinian Wu

**Affiliations:** 1 Institute of Grassland Research, Chinese Academy of Agricultural Sciences, Hohhot 010010, China; lishichao990920@163.com (S.L.);; 2 Pratacultural College, Gansu Agricultural University, Lanzhou 730070, China; 3 Key Laboratory of Grassland Resources and Utilization of Ministry of Agriculture, Hohhot 010010, China

**Keywords:** *Bromus*, repeat analysis, chloroplast genome, phylogenetic relationship, codon usage

## Abstract

*Bromus* (Poaceae Bromeae) is a forage grass with high adaptability and ecological and economic value. Here, we sequenced *Bromus ciliatus*, *Bromus benekenii*, *Bromus riparius*, and *Bromus rubens* chloroplast genomes and compared them with four previously described species. The genome sizes of *Bromus* species ranged from 136,934 bp (*Bromus vulgaris*) to 137,189 bp (*Bromus ciliates*, *Bromus biebersteinii*), with a typical quadripartite structure. The studied species had 129 genes, consisting of 83 protein-coding, 38 tRNA-coding, and 8 rRNA-coding genes. The highest GC content was found in the inverted repeat (IR) region (43.85–44.15%), followed by the large single-copy (LSC) region (36.25–36.65%) and the small single-copy (SSC) region (32.21–32.46%). There were 33 high-frequency codons, with those ending in A/U accounting for 90.91%. A total of 350 simple sequence repeats (SSRs) were identified, with single-nucleotide repeats being the most common (61.43%). A total of 228 forward and 141 palindromic repeats were identified. No reverse or complementary repeats were detected. The sequence identities of all sequences were very similar, especially with respect to the protein-coding and inverted repeat regions. Seven highly variable regions were detected, which could be used for molecular marker development. The constructed phylogenetic tree indicates that *Bromus* is a monophyletic taxon closely related to Triticum. This comparative analysis of the chloroplast genome of *Bromus* provides a scientific basis for species identification and phylogenetic studies.

## 1. Introduction

*Bromus* (Poaceae Bromeae) is an important forage grass with annual, biennial, and perennial growth [1]. It is mainly found in Europe, Asia, America, and Africa, and is highly adaptable to drought, salinity, and cold [2]. The majority of *Bromus* species are utilized as cereal crops, pasture grass, or silage, and some have important medicinal value [2,3,4]. This genus contains approximately 250 species [5], and the probability of polyploidy in these species is high, ranging from 2× to 12× [2]. Owing to its complex classification and wide geographical scope, no comprehensive classification exists of all *Bromus* species in the world; however, a number of regional descriptions and identification tables for *Bromus* plants have been published in different regions [6,7,8]. The criteria for *Bromus* classification remain debatable. *Bromus* is divided into six groups, five genera, and seven subgenera based on morphology, serology, and cytogenetics; however, reliance on shared morphological features may lead to taxonomic confusion because parallel or convergent evolution is widespread in the family Poaceae [9,10,11]. Pillay [12,13] studied the variation in the chloroplast (cp) genome enzyme cleavage sites, constructed a physical map of the cp genome and a simple phylogenetic tree, and confirmed that the cp genome is a useful tool in the study of *Bromus* phylogeny. A study by Saarela [14] revealed discrepancies between the results of plastid and ribosomal trees, indicating the need for additional data to define *Bromus* genetic relationships. Previous studies have formed the foundation for identifying and subdividing *Bromus*. However, the intricate evolutionary relationships remain uncertain.

Chloroplasts are semi-autonomous hereditary organelles formed by eukaryotic cells that phagocytose cyanobacteria [15,16]. They are important sites for energy conversion and photosynthesis in plants; are commonly found in algae, land plants, and protists; and synthesize proteins, starches, and fatty acids [17]. In most plants, the cp genome is double-stranded and has a typical quadripartite structure, including a large single-copy (LSC) region, small single-copy (SSC) region, and two copies of inverted repeat (IR) regions [18]. The sizes of plant cp genomes range from 107 to 218 kb, with variations primarily caused by contraction and expansion of the IR regions [19]. The cp genome is characterized by maternal inheritance, a moderate evolutionary rate [20], relatively stable genome content [21], and a low recombination rate [22,23]. In nature, there are a multitude of plant species, and identification of closely related species has always been a challenge [24]. With the advancement of sequencing technology, the cp genome has become a widely used molecular marker in systematic studies of diversity, phylogeny, and taxonomic issues, and it has become one of the most useful tools in molecular systematics [25]. Cp DNA sequence fragments, such as *matK*, *rbcL*, *trnH*, *psbA*, *rpoC1*, *rpoB*, *accD*, *ycf5*, and *ndhJ*, are commonly used for plant DNA barcoding [18]. Pillay [13] analyzed the enzyme cleavage sites of *Bromus* using phylogenetic information and obtained a simple phylogenetic chart, which provided a preliminary analysis of the genetic relationships of *Bromus*. Saarela and Nasiri [1,14] confirmed the monophyletic nature of *Bromus*. However, the ribosomal and cp data were inconsistent for the *Bromus* major lineage. Yoshihiro et al. [26] performed a comparative analysis of rice, maize, and wheat cp genomes in Poaceae, focusing mainly on non-coding regions, and constructed a grass phylogenetic tree. To date, more than 4671 species of Poaceae plants have been sequenced, including nine species of *Bromus* plants. Nevertheless, the number of publicly available whole-genome *Bromus* sequences remains relatively limited, despite the crucial role of the cp genome in classification.

Previous studies have analyzed the classification, genetic relationships, and phylogeny of *Bromus* based on morphology and molecules; however, few analyses have been conducted on the basis of the cp genome, and no comprehensive and systematic studies of *Bromus* have been conducted. In this investigation, we conducted sequencing of the cp genomes of four *Bromus* species and conducted analysis in conjunction with previously published data from four additional sequenced species (*B. inermis*, *B. biebersteinii*, *B. vulgaris*, and *B. diandrus*), with the goal of gaining insights into the phylogenetic relationships between these taxa. Furthermore, we examined boundary stretching, nucleotide polymorphisms (pi), simple repeats (SSRs), codon usage bias, and the phylogeny of eight *Bromus* plants. The aim of this study was to identify *Bromus* species and understand the phylogenetic relationships between these and other *Bromus* species.

## 2. Materials and Methods

### 2.1. Genome Sequencing, Assembly, and Annotation

Following retrieval from the field, four *Bromus* seeds were deposited at the National Intermediate Forage Germplasm Bank in Hohhot, China (40.57° N, 111.93° E). Utilizing the TIANamp Genomic DNA Kit (Tiangen Biotech Co., Ltd., Beijing, China), genomic DNA concluding chloroplast DNA or genomic DNA were isolated from fresh leaves. Subsequently, next-generation sequencing took place on the MiSeq PE150 platform, yielding 150 bp paired reads. Only the cp genome was assembled with GetOrganelle v1.7.7.0 [27] and annotated employing the Plastid Genome Annotator tool [28]. Any inaccuracies in the identification of initiation and stop codons by the Plastid Genome Annotator were rectified manually using Geneious v9.0.2 [29,30]. The cp genome sequences of *B. inermis* (MW861351.1), *B. biebersteinii* (MW309816.1), *B. vulgaris* (NC_027472.1), and *B. diandrus* (NC_082233.1) were acquired from GenBank.

### 2.2. Identification of Repeat Sequences and SSR Sequences

Different repetition types with a minimum repeat length of 30 and a Hamming distance of 3 [31], including complementary, palindromic, forward, and reverse types, were identified by REPuter tools. MISA software v2.1 was used for the SSRs detected [32], with parameter configurations delineating the unit size (in nucleotides) and minimum repeat: 1-10, 2-6, 3-4, 4-3, 5-3, and 6-3. Finally, the minimum interspace between two SSRs was set as 100 base pairs.

### 2.3. Comparative Genome Analysis

Variations among all *Bromus* cp genomes were compared using mVISTA [33], with *B. inermis* as the reference sequence. Additionally, IR-Scope was used for the comparison and visual representation of the connections and boundaries within the IR regions [34]. The cp genomes of all eight *Bromus* species were aligned using MAFFT v7.313 [35] with default settings. Nucleotide diversity (Pi) rates, indicating sequence divergence among *Bromus* species, were then calculated using DnaSP v6.12 [36].

### 2.4. Phylogenetic Analysis

For the phylogenetic analysis, we employed the entire cp genomes and common protein-coding genes of the four recently sequenced *Bromus* species, along with sequences from four *Bromus* species and two outgroup species (*Oryza sativa* (NC_008155), *Brachypodium distachyon* (NC_011032)) previously documented in the NCBI database (Table S1), as well as 27 species from 10 genera in Poaceae. The sequences were aligned by MAFFT v7.313 with default parameters [35] and used for constructing trees via both the maximum likelihood (ML) and Bayesian inference (BI) methods [37]. The shared protein-coding genes were analyzed using the GTR + F + I + G4 substitution model for both the maximum likelihood (ML) and Bayesian inference (BI) methods. The ML analysis was conducted with RAxML v8.2.11 based on a non-parametric bootstrap approach with 1000 replicates [38], while the Bayesian inference analysis was conducted with MrBayes v3.2.6 software [39].

## 3. Results

### 3.1. Genomic Features of Bromus

**Figure 1:**
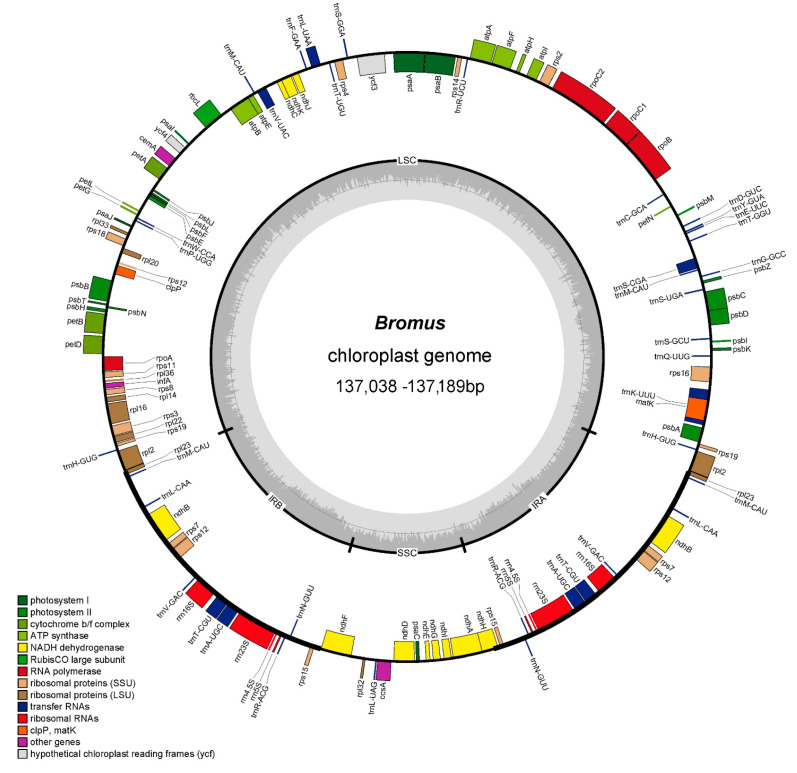
Circular diagram of the *Bromus* chloroplast genome. Genes depicted within the circumference are transcribed in a clockwise direction, while those situated outside are transcribed counterclockwise. Genes affiliated with distinct functional categories are colorized and labeled accordingly in the accompanying key (LSC: large single-copy, SSC: small single-copy, IR: inverted repeat).

**Figure 2:**
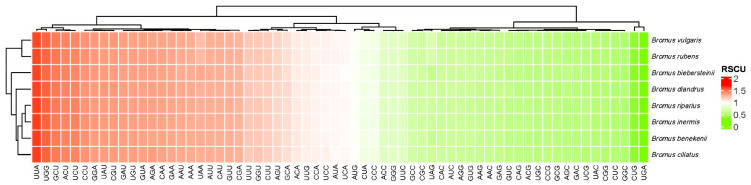
The relative synonymous codon usage (RSCU) values of eight *Bromus* species.

The cp genome of the genus demonstrates the typical quadripartite structure (Figure 1), where the lengths of the LSC, SSC, and IR regions differ among species, spanning from 81,128 bp (*B. rubins*) to 85,638 bp (*B. riparius*) for LSC, 12,610 bp (*B. benekenii*) to 12,640 bp (*B. rubens*) for SSC, and 19,410 bp (*B. benekenii*) to 21,706 bp (*B. rubens*) for IR (Table S2). The GC contents of the newly sequenced cp genomes vary between 38.31% and 38.38%, encompassing the GC content of all recognized *Bromus* species (Table S2). The highest GC content was found in the IR region (43.85–44.15%), followed by the LSC region (36.25–36.65%) and the SSC region (32.21–32.46%). In all, 64 different codons, which encode for 21 amino acids, were detected in all *Bromus* sequences (Figure 2).

The cp genomes of the four *Bromus* species ranged in size from 137,038 bp (*B. benekenii*) to 137,189 bp (*B. ciliatus* and *B. biebersteinii*). They comprised a total of 129 genes, consisting of 83 protein-coding genes, 38 tRNA-coding genes, and 8 rRNA-coding genes. These genes constituted approximately 64.34%, 29.46%, and 6.20% of all genes, respectively (Figure 1, Table S2). Among the genes, five related to photosynthesis and eight related to self-replication had introns. One ribosomal small subunit (*rps12*) gene and one with an unknown function (*ycf3*) had two introns. Four rRNAs (*rrn16S*, *rrn5S*, *rrn4.5S*, and *rrn23S*), seven tRNAs (*trnL-CAA*, *trnH-GUG*, *trnA-UGC*, *trnN-GUU*, *trnT-CGU*, *trnR-ACG*, and *trnV-GAC*) and seven protein-coding genes (*rpl23*, *rps19*, *rps15*, *rpl2*, *rpl12*, *rps7*, and *ndhB*) had two copies. In addition, a four-copy tRNA gene (*trnM-CAU*) was found (Table 1).

The total number of codons in the new sequences was found to be 19,696–19,872. AUU was the most abundant (821–838) and UGA was the least (16–25) (Figure 2). Within the *Bromus* cp genome, the relative synonymous codon usage (RSCU) values ranged from 0.09 to 2.09 for all codons, with 33 codons exhibiting a high frequency, possessing RSCU values exceeding 1.00. Of the codons exhibiting RSCU values greater than 1, 90.91% concluded with A/U bases, while 9.09% concluded with C/G bases. Codon UUA, which encodes phenylalanine (Phe), had the highest relative probability of use, with an RSCU of 2.09. The RSCU values of the remaining 32 codons ranged from 1.01 to 1.91. This analysis revealed a preference for codons ending in A/U in the *Bromus* cp genome.

### 3.2. Repeat Analysis

**Figure 3:**
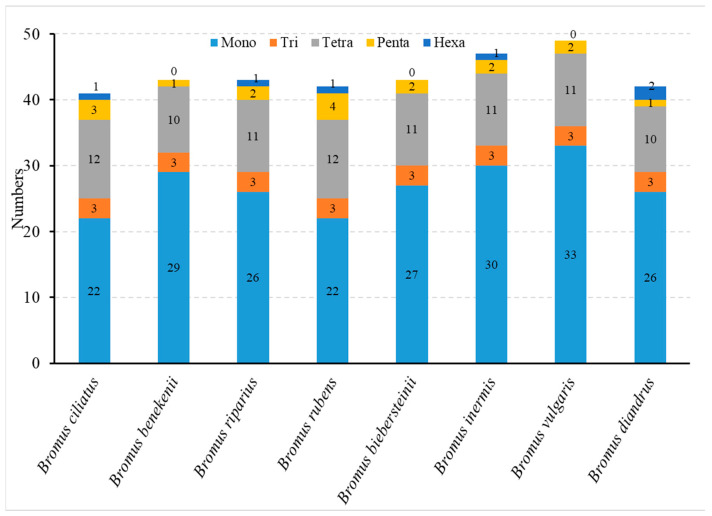
Analysis of diverse SSR variants across eight *Bromus* chloroplast genomes.

In total, 350 SSRs were identified across eight genomes of members of the *Bromus* genus (Figure 3). The majority of the SSRs consisted of A/T pairs rather than C/G pairs. The quantity of SSRs in *Bromus* varied between 41 and 49. Single-nucleotide repeats constituted the most prevalent type of repetition (61.43%), succeeded by tetranucleotide repeats (25.14%) and trinucleotide repeats (6.9%). Notably, the *Bromus* cp genome lacks dinucleotide repeats. Among the studied species, only *B. ciliatus*, *B. riparius*, *B. rubens*, and *B. inermis* had a single hexanucleotide repeat sequence, whereas *B. diandrus* had two hexanucleotide repeat sequences. The AATTAG/AATTCT repeats were detected in *B. ciliatus* and *B. rubens*, whereas the AATCCT/AGGATT repeats were detected in both *B. riparius* and *B. inermis*. In addition, *B. diandrus* contained both the AAAAAT/ATTTTTT and ACATCT/AGATGT repeats (Table S3).

**Figure 4:**
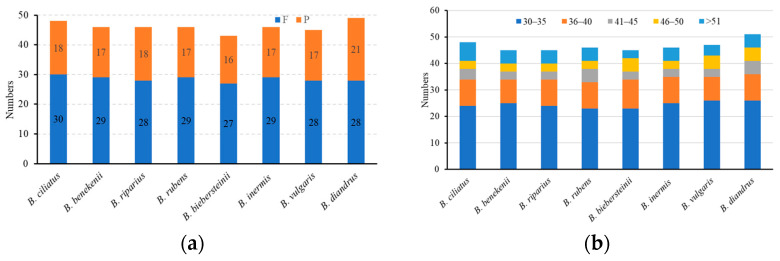
Tandem repeats sequences in the eight *Bromus* chloroplast genomes. (**a**) Total number of repeat types, F: forward repetitive sequences, P: palindromic repetitive sequences. (**b**) Number of repeats by length.

Long repetitive sequences are repetitive sequences ≥30 bp in length, which are favorable for genome rearrangement and increase population genetic diversity. A total of 369 repetitive sequences were detected in *Bromus*, including 228 forward (F) and 141 palindromic (P) repetitive sequences; no reverse (R) or complementary (C) repetitive sequences were detected (Figure 4). The lowest number of repeats was found in *B. biebersteinii* (43), and the highest was found in *B. diandrus* (49). *B. rubens* and *B. diandrus* had a higher number of repeats (at 41–45 bp) than those in the other six species. *B. biebersteinii*, *B. vulgaris*, and *B. diandrus* had a higher number of repetitive sequences (46–50 bp) that were repeated five times, whereas the other species showed repeats of only three times.

### 3.3. Comparative Analysis of the Bromus Chloroplast Genome

**Figure 5:**
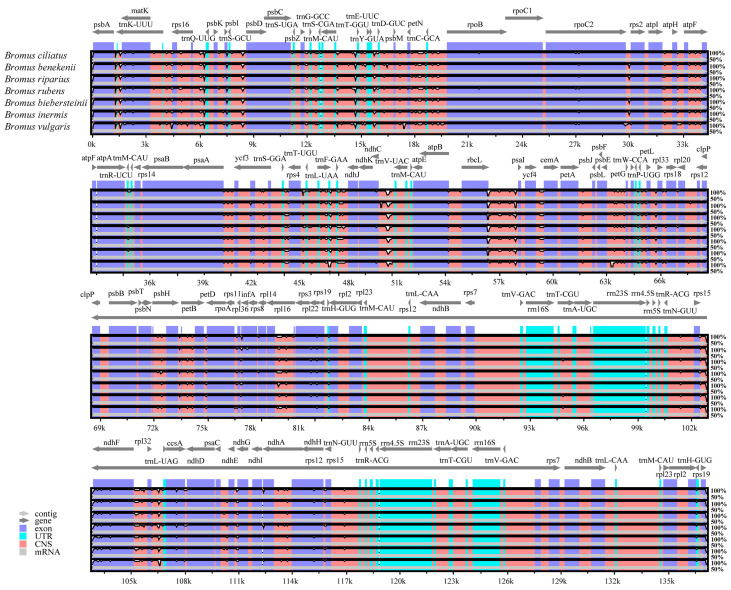
Eight chloroplast genomes were aligned globally using *B. inermis* as a reference. Each coordinate represents a region of the chloroplast genome. The vertical axis represents the percentage of the aligned region sequence similarity.

Regions of significant variability within cp genomes serve as valuable tools for discerning closely related species and conducting molecular evolution investigations [40]. Using *B. inermis* as a benchmark, mVISTA was employed to analyze the cp genomes of seven *Bromus* species, incorporating both newly sequenced genomes and those retrieved from the NCBI repository, in order to explore sequence diversity (Figure 5). The cp genome is homologous within *Bromus*, and is conserved across species with high covariance; however, there are some differences. The mutation rate varied in different regions, which reflects the evolution of Bromus cps. There were differences in cp genome lengths between species, with *B. vulgaris* being the shortest (136,934 bp) and *B. ciliatus* and *B. biebersteinii* being slightly longer (137,189 bp). Overall, the cp genomes of the other seven *Bromus* species displayed notable structural similarity and maintained a high level of conservation regarding both size and gene sequences. However, the number of some conserved non-coding region (CNS) variants, including *rpoC2*-*rps2*, *ndhC*-*trnV*-*UAC*, *psbM-petN*, *trnF-GAA-ndhJ*, *psbE-petG*, and *rpl16*, was higher than those in other regions. In the non-coding region between *rpoC2* and *rps2*, *B. ciliatus* and *B. rubens* showed no mutations. *B. benekenii*, *B. riparius*, *B. biebersteinii*, and *B. inermis* had similar mutations, and *B. vulgaris* had mutations that differed from those in the other six species. In the non-coding region between *atpB* and *rbcL*, *B. ciliatus* and *B. rubens* had similar variations that differed from those in the other five species. No variations were found in *B. ciliatus* and *B. rubens* in the exonic region of *ycf3*, while the remaining five species showed similar variations.

**Figure 6:**
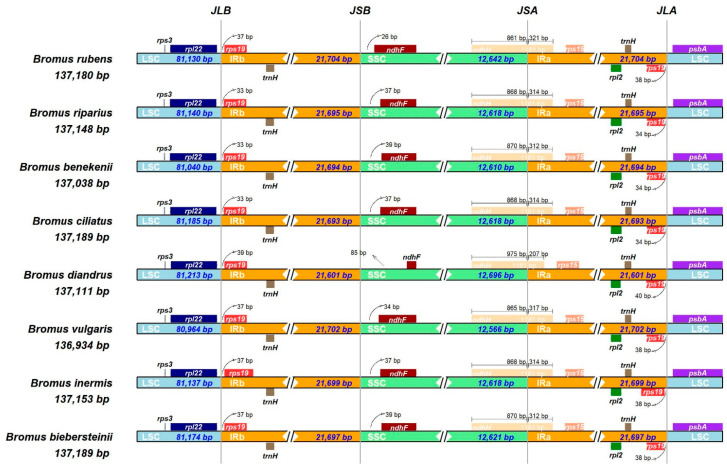
Comparison between LSC, IR, and SSC junction boundaries in the cp genomes of eight *Bromus* species. JLB, JSB, JSA, and JLA are representative of LSC/IRb, SSC/IRa, LSC/IRa, and LSC/IRa, respectively.

The intergenic spacer (IR) region of the cp genome is considered highly conserved; however, its boundary region can contract or expand, causing the cp genome to change in length [41]. Therefore, we investigated the structural features of the LSC, SSC, and IR regions in the cp genomes of the eight Bromus species. Specifically, we focused on the LSC/IRb junction (JLB), positioned between the *rpl22* gene within the LSC region and the *rps19* gene within the IRb region (Figure 6). It is noteworthy that the *rps19* gene spans from the IRb into the LSC, typically ranging from 33 to 39 bp in length. The boundary known as JLA, which separates the LSC and IRa regions, is positioned between the *psbA* gene in the LSC and the *rps19* gene in the IRa. The *rps19* gene crosses from the IRa into the LSC, covering a distance of 34–40 bp. Furthermore, the *ndhF* gene is positioned within a range of 26 to 85 bp from the boundary of IRb and SSC (JSB). In all Bromus species, the *ndhH* gene extends across the junction (JSA) between the SSC and IRa regions, with lengths varying from 861 to 975 bp in the SSC region and 207 to 321 bp in the IRa region. The variations in the lengths of these regions primarily manifest in the positions of *rps19*, *rpl22*, *ndhF*, *ndhH*, and *psbA*, indicating dynamic expansions and contractions in the inverted repeat regions.

**Figure 7:**
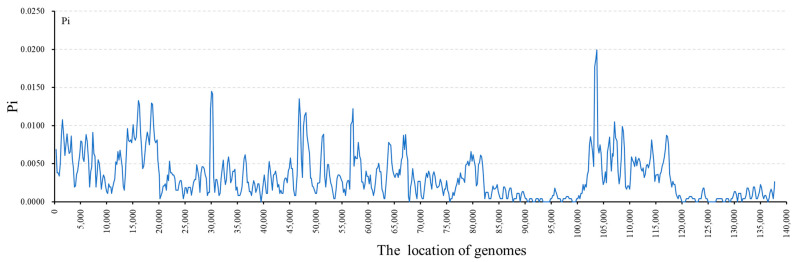
Nucleotide diversity (Pi) values of eight *Bromus* species shown as a line chart. On the X-axis, locations of cp genomes are listed. The values of Pi are shown on the Y-axis.

The eight *Bromus* species were identical to the others. The most conserved region was the IR region, with only a few diversity hotspots. The bulk of the areas exhibiting high genetic diversity were situated within the LSC and SSC regions (Figure 7). The peak Pi value within the notably variable region reached 0.01994, while the nadir was noted at 0.01006. Twenty-two highly mutated regions with Pi values greater than 0.01 were detected, namely, *trnC-GCA*, *trnT-GGU*, *rbcL-psal*, *trnL-UGA-ccsA*, *rbcL-psal*, *psbA-matK*, and *trnD-GUC*, *trnT-UGU-trnL-UAA*, *trnL-UAA-trnF-GAA*, *trnL-UAA-trnF-GAA*, *rpoC2-rps2*, *rbcL-psal*, *trnD-GUC-psbM*, *trnC-GCA*, *petN-trnC-GCA*, *trnD-GUC-psbM*, *trnT-UGU-trnL-UAA*, *rpoC2-rps2*, *rpoC2-rps2*, and three *ndhF*. These regions exhibit high levels of mutation, making them ideal for phylogenetic analyses of *Bromus* species and the development of molecular markers for plant identification.

### 3.4. Phylogenetic Analysis

**Figure 8:**
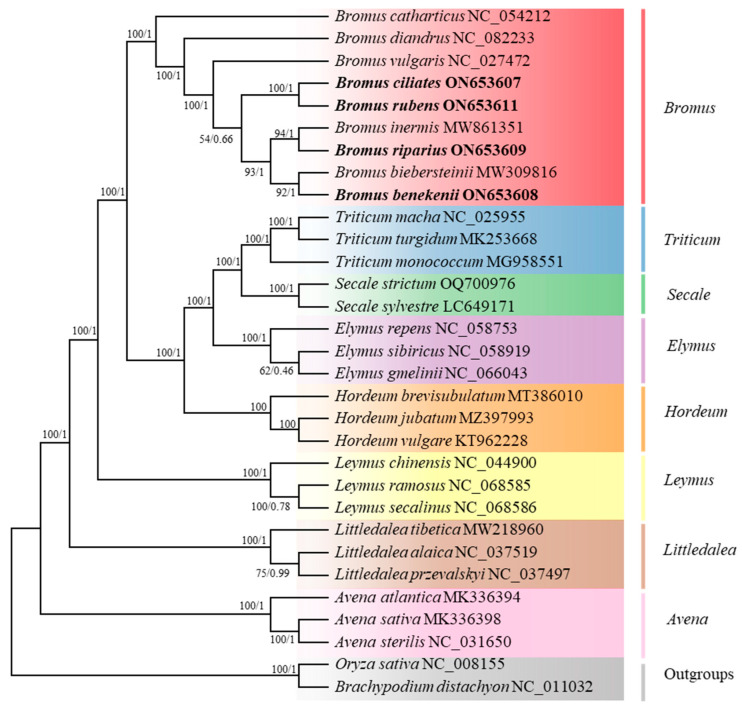
Phylogenetic tree of *Bromus* with 21 other representative Poaceae Barnhart species. *B. distachyon* and *O. sativa* were selected as the outgroups. The maximum likelihood (ML) and Bayesian inference (BI) methods were used to construct the tree based on shared protein-coding genes. Maximum likelihood bootstrap support values and Bayesian posterior probabilities are shown for each node.

For clarification of the affinities and phylogenetic positions of *Bromus* during the evolutionary process (Figure 8), we downloaded the cp genome sequences of 27 species from 10 genera from the NCBI database. A phylogenetic tree was constructed using *O. sativa* and *B. distachyon* as outgroups. The topology of the tree revealed that the 8 *Bromus* species clustered in different branches from the 21 species of the other seven Poaceae. In the *Bromus* branch, *B. catharticus* first diverged to form a branch independent of the other *Bromus* species, followed by *B. diandrus* and *B. vulgaris*. The remaining six species were shown to be closely related to each other. Among these, *B. inermis* and *B. riparius*, as well as *B. biebersteinii* and *B. benekenii*, clustered together and were the most closely related.

## 4. Discussion

In this investigation, we sequenced, annotated, compared, and analyzed the complete cp genomes of *B. ciliates*, *B. benekenii*, *B. riparius*, and *B. rubens*, alongside four previously reported *Bromus* cp genomes. The cp genomes of all eight *Bromus* species had a typical quadripartite structure consisting of LSC, SSC, IRa, and IRb, with full genome lengths ranging from 137,038 bp (*B. benekenii*) to 137,189 bp (*B. ciliatus*) (Table S3 and Figure 1), which is in agreement with published data on *Bromus* species [12,42,43]. Compared to cattail (*Typha orientalis presl*) and tobacco (*Nicoticular tabacum* L.), the genes *accD*, *ycf1*, and *ycf2* have undergone progressive degradation and eventual loss in the *Bromus* cp genome [44]. This phenomenon has also occurred in other Poaceae species, such as *Cynodon dactylon* [45,46]. A possible reason for the elimination of *accD*, *ycf1*, and *ycf2* may be their lack of a significant advantage for survival or reproduction, followed by natural selection. Alternatively, this may be attributed to shifted-code mutations and gene deletions caused by insertions and deletions of non-triplet bases [44]. We detected a four-copy tRNA gene (*trnM-CAU*) in the *Bromus* cp genome. Sutton [47] found that an increase in boron toxicity tolerance in barley was due to an increase in the copy number of boron transporter proteins. Würschum [48] identified the role of copy number variation in *Ppd-B1* and *Vrn-A1* in global wheat adaptation. Copy number variations may lead to the generation of new gene functions, changes in gene expression levels, and reorganization of gene interaction networks, which may have an impact on the structure, function, and adaptation of polyploid plants, which played key roles in the evolution, adaptation, and gene function of many species [49]. RSCU values >1 indicate a preference for using the codon, whereas RSCU values <1 indicate the opposite [50].Codon preference exists in most plant genomes, and natural selection and mutation pressures are the main factors that affect this preference [51]. In the present study, we identified 264 RSCUs value > 1 and 248 RSCU values < 1 in the cp genomes of *Bromus*. Among the codons with RSCU values greater than >1, 90.91% of them ended with base A/U and 9.09% with C/G. This codon preference for the use of A/U endings is similar to that of found in other Poaceae species [52].

SSRs have been widely used as important genetic markers in genetic, polymorphic, and evolutionary studies on plant populations [53,54]. In total, 350 SSR sequences were detected in the eight *Bromus* cp genomes (Figure 3). These sequences demonstrated the highest A/T content at 58.6% and the greatest number of repeats, aligning with findings reported in other plant studies [55,56,57]. The repeat units AAAAAT/ATTTTTT and ACATCT/AGATGT, unique to *B. diandrus*, and the repeat unit AAAAT/ATTTTT, unique to *B. ciliatus*, discovered in this study can serve as molecular markers to differentiate between the two species. These markers can enhance our understanding of the population structure and genetic diversity of these species, and they will be crucial for molecular breeding and genetic engineering [58].

The expansion and contraction of IR regions along with single-copy boundaries are pivotal in plant cp genome evolution [59]. Comparative sequence analysis coupled with IR boundary examination revealed highly consistent gene arrangement lengths and orders across the cp genomes of these eight plant species. Nonetheless, discrepancies arose in boundary gene types and positions, with the most significant variations occurring in the SSC/IR boundaries. These differential sequences provide new potential resources for identifying and studying *Bromus* species. Additionally, comparative sequence analysis revealed that the CNS region had greater variation than other regions, which is consistent with findings in other *Bromus* species [60,61]. The smallest reverse IR region was identified in *B. benekenii* (19,410 bp), and the largest reverse IR region was found in *B. rubens* (21,706 bp). This difference could be attributed to the expansion and contraction of the IR region, along with the impact of the single-copy spacer region, resulting in variations in the overall length of the cp genome [62]. The *ndhH* gene was also found at both ends of the JSA boundary, consistent with previously reported findings in other Poaceae species [46,63,64,65]. The different locations of this gene at the JSA boundary may be attributed to the intramolecular recombination during early evolution [66].

The findings of our phylogenetic analyses supplement insights from earlier investigations, indicating the potential utility of the cp genome in elucidating inter-species relationships within the genus. In most studies, Bromeae and Triticeae are sister groups; however, studies based on the whole Bromeae plastome are nested in Triticeae, making Triticeae paraphyletic [67,68]. The results of this study support *Bromus* and *Triticum* as sister groups. *B. inermis*, *B. benekenii*, and *B. riparius*, which all belong to *Bromopsis*, constitute several independent lineages and are not clustered into a single clade, which is in agreement with the results of Saarela and Pillay [13,14]. Nasiri [1] found that *Bromeae sect. Genea* is not monophyletic in plastid trees. In the present study, *B. rubens* and *B. diandrus*, both belonging to *B. sect. Genea*, were not clustered into a single lineage but were sister groups. The cp DNA evolutionary tree constructed by Pillay [13] showed that *Ceratochloa* is uniquely characterized and separated from other genera, including *Bromus*. In summary, *B. catharticus* belongs to *Ceratochloa*, forming a separate clade. This may be because it emerged in the early Pliocene, earlier than the origin of other *Bromus* subgenera.

## 5. Conclusions

In the examination of four recently sequenced and four previously documented *Bromus* cp genomes, it was observed that these genomes exhibit a high level of conservation and share a similar overall structure across the genus. Moreover, our investigation highlighted the prevalence of A/T bases in SSR sequences and revealed distinctive repeat units. High similarity in the cp genome sequences was observed among *Bromus* species, particularly in the coding and IR regions. Seven highly variable CNS regions were pinpointed as potential novel genetic markers for DNA barcoding and molecular phylogenetic analyses. Through a comparative examination of IR boundaries, unique genomic placements for *rpl22*, *rps19*, *ndhH*, and *psbA* were identified, attributed to IR contraction and expansion events in *Bromus* species. Notably, the establishment of *Bromus* as a monophyletic taxon closely related to *Triticum* was highlighted. These discoveries offer valuable insights into both the identification and phylogenetic classification of *Bromus* species.

## Figures and Tables

**Table 1:** Gene composition in the *Bromus* chloroplast genome.

Category	Group	Name
Photosynthesis	Photosystem I	*psaA*, *psaB*, *psaC*, *psaI*, *psaJ*
	Photosystem II	*psbA*, *psbB*, *psbC*, *psbD*, *psbE*, *psbF*, *psbH*, *psbI*, *psbJ*, *psbK*, *psbL*, *psbM*, *psbN*, *psbT*, *psbZ*
	NADH-dehydrogenase	ndhA*, ndhB*(2), *ndhC*, *ndhD*, *ndhE*, *ndhF*, *ndhG*, *ndhH*, *ndhI*, *ndhJ*, *ndhK*
	Cytochrome b/f complex	*petA*, petB*, petD*, *petG*, *petL*, *petN*
	ATP synthase	*atpA*, *atpB*, *atpE*, atpF*, *atpH*, *atpI*
	Rubisco	*rbcL*
Self-replication	Large subunit of ribosome	*rpl14*, rpl16*, rpl2*(2), *rpl20*, *rpl22*, *rpl23(2)*, *rpl32*, *rpl33*, *rpl36*
	Small subunit of ribosome	*rps11*, rps12**(2), *rps14*, *rps15(2)*, rps16*, *rps18*, *rps19(2)*, *rps2*, *rps3*, *rps4*, *rps7(2)*, *rps8*
	DNA dependent RNA polymerase	*rpoA*, *rpoB*, *rpoC1*, *rpoC2*
	rRNA genes	*rrn16S(2)*, *rrn23S(2)*, *rrn4.5S(2)*, *rrn5S(2)*
	tRNA genes	trnA-UGC*(2), *trnC-GCA*, *trnD-GUC*, *trnE-UUC*, *trnF-GAA*, *trnG-GCC*, *trnH-GUG(2)*, trnK-UUU*, *trnL-CAA(2)*, trnL-UAA*, *trnL-UAG*, *trnM-CAU(4)*, *trnN-GUU(2)*, *trnP-UGG*, *trnQ-UUG*, *trnR-ACG(2)*, *trnR-UCU*, trnS-CGA*, *trnS-GCU*, *trnS-GGA*, *trnS-UGA*, trnT-CGU*(2), *trnT-GGU*, *trnT-UGU*, *trnV-GAC(2)*, trnV-UAC*, *trnW-CCA*, *trnY-GUA*
Other genes	C-type cytochrome synthesis gene	*ccsA*
	Envelop membrane protein	*cemA*
	Protease	*clpP*
	Acetyl-CoA carboxylase	*infA*
	Maturase	*matK*
Unknown	Conserved open reading frames	ycf3**, *ycf4*

Notes: *, intron number; ** the number in the bracket signifies the number of copies of the gene.

## Data Availability

The complete chloroplast genome sequences were deposited at NCBI with different GenBank accession numbers: ON653607, ON653608, ON653609, and ON653611.

## Supplementary Materials

The following supporting information can be downloaded at https://www.mdpi.com/article/10.3390/genes15060815/s1, Table S1: GenBank accession numbers of chloroplast genomes; Table S2: Summary of chloroplast genome characteristics in *Bromus* species; Table S3: Numbers of identified SSRs motifs in the 8 *Bromus* chloroplast genomes.
